# Psoriasis: Female Skin Changes in Various Hormonal Stages throughout Life—Puberty, Pregnancy, and Menopause

**DOI:** 10.1155/2013/571912

**Published:** 2013-12-28

**Authors:** Romana Ceovic, Marko Mance, Zrinka Bukvic Mokos, Maja Svetec, Kresimir Kostovic, Daska Stulhofer Buzina

**Affiliations:** ^1^Department of Dermatology and Venereology, Zagreb University Hospital Center and School of Medicine, 10 000 Zagreb, Croatia; ^2^Ministry of Justice, 10 000 Zagreb, Croatia; ^3^Institute of Emergency Medicine, 42 000 Varazdin County, Croatia

## Abstract

Psoriasis is one of the most prevalent immune mediated skin diseases worldwide. Despite the large prevalence in both men and women, the pathogenesis of this disease has not yet been fully clarified. Nowadays, it is believed that psoriasis is most likely a T helper Th1/Th17 induced inflammatory disease. Stressful life situations are known to cause flare-ups and psoriasis activity may be linked to stress from major life events. We know that stress greatly affects both the hormone and immune systems and that there are many different hormonal phases throughout a woman's lifetime. The severity of psoriasis may fluctuate or be influenced by each phase and this relationship can be seen as disease frequency seems to peak during puberty, postpartum, and menopause when hormone levels fall, while symptoms improve during pregnancy, a state when hormone levels are increased.

## 1. Introduction

Psoriasis affects approximately 25 million people in North America and Europe and is one of the most prevalent immune mediated skin diseases in adults [1]. It is a chronic, inflammatory skin disorder characterized by erythematous, scaly patches, and plaques that can affect any part of the body [2]. The incidence of psoriasis is similar in male and female populations, with the mean age of presentation in females at 26.94 ± 14.94 years [3].

The pathogenesis of psoriasis is considered to be an immune mediated process that takes place upon a favorable genetic background. The presence of a yet unknown (auto)antigen causes the generation of effector T-cells that infiltrate the skin and initiate the inflammatory process [4]. The disease pathogenesis is linked to many interactive responses among infiltrating leukocytes, resident skin cells, and an array of proinflammatory cytokines, chemokines, and chemical mediators produced in the skin [5]. Nowadays, it is believed that psoriasis is most likely a T helper Th1/Th17 induced inflammatory disease [6].

Psoriasis is associated with metabolic syndrome and the association increases with increasing disease severity [7]. The impact of psoriasis on the patient quality of life is similar to that in patients living with insulin-dependent diabetes, depression, and cardiovascular diseases [8]. Various environmental risk factors, including trauma to the skin, infections, obesity, smoking, alcohol use, emotional stress, and various drugs, have been associated with psoriasis. Disease flare-ups are known to occur in stressful life situations and certain literature links psoriasis activity to stress from major life events [9]. The exact mechanism of how psoriasis is induced or aggravated is not known, but we do know that stress greatly affects both the hormone and immune systems [10, 11]. There are many different hormonal phases throughout a woman's lifetime and the first symptoms of psoriasis can be seen at any time, that is, in childhood, menarche, pregnancy, during hormonal contraceptive use, and menopause [12]. The severity of psoriasis may fluctuate or be influenced by each phase and this relationship can be seen as disease frequency seems to peak during puberty, postpartum, and menopause, when hormone levels fall, while symptoms improve during pregnancy, a state when hormone levels are increased [13, 14]. Recent studies have shown that female hormones significantly affect the biological and immune changes in the skin [15]. The Th2 immune response, shown to be potentiated by estrogen, results in increased concentrations of interleukin (IL)-4, IL-5, and IL-10, while androgens have been found to increase the Th1 response leading to increased IL-2 production and activation of CD8+ T-cells. Interestingly, these differences are marked in reproductive years but disappear after menopause [16].

## 2. Psoriasis during Puberty

Psoriasis in childhood and adolescence is not uncommon and many studies indicate the appearance of psoriatic lesions by 16 years of age in one-third of patients [17]. It has been observed that 63.5% of patients develop the disease before the age of 30 [18]. The age at onset was documented by Swanbeck et al. to begin around puberty as well as between the ages of 30 and 50 [19]. This bimodal age at onset has also been described by Henseler and Christophers, who report on the mean age at onset of psoriasis presentation to range between 15 and 20 years of age in 2,147 patients, with a second peak occurring at the ages of 55–60 [20]. A relationship between psoriasis and hormonal changes in different stages of life has been observed; however, it has not yet been identified [21]. In women, hormonal changes such as those that occur at puberty can trigger or worsen psoriasis, which has also been mentioned by Islam et al. [3, 22]. Murase et al. also observed a relationship between the development of psoriasis and puberty and found that many people developed their first psoriatic lesions just after puberty, which correlates with the decrease in hormone levels [13]. A recent study by Kanda and Watanabe has revealed that sex hormones manifest a variety of biological and immune effects in the skin and that hormone fluctuations during a woman's menstrual cycle can affect psoriasis [15]. During menstrual cycle, the follicle within the ovary is actively secreting estrogens until their serum levels reach the threshold value. After approximately 10 days, corpus luteum begins to degenerate, with estrogen and progesterone concentrations declining at around day 26 of the cycle. Luteinizing hormone (LH) level begins to rise and the follicles are therefore stimulated to mature, so that by the beginning of the new cycle, estrogen levels are once again on the rise [21]. Kanda and Watanabe have found that menstruation is associated with modulation of the natural course of psoriasis, suggesting that skin inflammation may be hormone induced. It has been observed that estrogen downregulates the production of the neutrophil, T-cells, and macrophage-attracting chemokines, CXCL8, CXCL10, and CCL5, by keratinocytes, and suppresses IL-12 production and antigen-presenting capacity while enhancing anti-inflammatory IL-10 production by dendritic cells, indicating how inflammation in psoriatic lesions may be linked to estrogens [15].

Increased levels of sex hormones, in particular estrogens, which are known to promote keratinocyte proliferation via specific receptor-mediated mechanisms, may explain the perimenarchal increase in the prevalence of psoriasis [23–28]. This mechanism appears to be significant in the wound-healing process, suggesting that this effect alone may provide a significant stimulus to the development of epidermal hypertrophy characteristic of psoriasis [29, 30].

Sex hormones are also known to influence inflammation [15, 31]. The increased levels of estrogen at menarche may influence the Th1 and Th2 immune responses through cytokines and chemokines, including monocyte chemoattractant protein-1 (MCP-1) production [23, 32, 33]. These changes may stimulate both the cellular activity and tumor necrosis factor (TNF)-alpha-induced inflammatory response, potentially providing a more direct link to the pathophysiology of psoriasis [23, 34, 35].

Generally, it can be assumed that high levels of estrogens seem to have a rather regulatory and inhibiting effect on many components of the immune response, while low levels can be stimulating [32, 36, 37]. These various regulatory effects of sex steroids and their fluctuations during puberty and adolescence have been linked to many skin conditions including psoriasis and are the focus of many therapeutic or prophylactic measures [15]. It is important for the patient as well as the physician to realize that psoriasis is a chronic condition and that hormonal changes can influence the course of the disease. Children, parents, and teenagers need to realize that psoriasis may recur throughout the patient's life and that it is of utmost importance to understand the disease and the hormonal phases that may aggravate it [22].

## 3. Pregnancy

Psoriasis affects women of all ages including reproductive years [38]. Estrogen and progesterone levels steadily increase throughout pregnancy until antepartum period [19]. Mowad et al. report on patients taking high-dose estrogen oral contraceptives and showing general improvement of their psoriasis, whereas Vessy et al. in their study in 92 patients concluded that there was little evidence for any effect of oral contraceptive use on psoriasis [39, 40]. Boyd and King found a different correlation when observing a patient whose psoriasis responded favorably to the administration of the antiestrogen compound tamoxifen [41].

During a woman's reproductive years, stressful life events such as pregnancy characterized by multiple physiologic changes influence the development of psoriasis and affect its clinical expression [3, 42]. Sex hormones, especially estrogen and prolactin (PRL), have an important role in modulating the immune response. Prolactin secreted from the pituitary gland as well as other organs and cells has an immune stimulatory effect and promotes autoimmunity. It interferes with B-cell tolerance induction, enhances proliferative response to antigens and mitogens, and increases the production of immune globulins, cytokines, and autoantibodies. Patients with hyperprolactinemia (HPRL) present with many different clinical manifestations, one of them being psoriasis. There are data indicating a correlation between PRL levels and disease activity [43]. Dhabhar has documented a connection between stress-related neurotransmitters, hormones, and other factors and exacerbation of certain immunopathologic conditions such as psoriasis [44]. Enhanced vascular endothelial growth factor (VEGF) production in macrophages is stimulated by estrogen, an effect that is antagonized by androgens, and it is believed that imbalance in hormone ratios could be related to the development of dermatologic diseases during pregnancy [13, 15, 44]. Oumeish and Al-Fouzan recognized the potential role of sex hormones in the etiology of psoriasis, since pregnancy, a state of natural immunomodulation, is associated with alleviation or exacerbation in various inflammatory diseases, including psoriasis [45, 46].

In their study, comparing hormonal effect on psoriasis in pregnancy, Murase et al. report that, during pregnancy, 55% of patients experienced improvement, 21% no change, and 23% worsening, while, postpartum, only 9% of patients experienced improvement, 26% no change, and 65% worsening. They found that psoriatic body surface area (BSA) significantly decreased from the 10th to the 20th week of gestation (*P* < 0.001) when compared with controls, whereas BSA significantly increased by 6 weeks postpartum (*P* = 0.001). Furthermore, in patients with 10% or greater psoriatic BSA, who reported improvement (*n* = 16; mean BSA, 40%), lesions decreased by 83.8% during pregnancy. They found that there were significant or near significant correlations between improvement in BSA and concentrations of certain hormones such as estradiol (*P* = 0.009; *r* = 0.648), estriol (*P* = 0.06; *r* = 0.491), and the estrogen to progesterone ratio (*P* = 0.006; *r* = 0.671). Therefore, it was concluded that high levels of estrogen correlated with an improvement in psoriasis, whereas progesterone levels did not correlate with psoriatic change [13]. Many studies investigated the relationship between hormones and psoriasis; it has been observed that worsening of symptoms occurs when estrogen and progesterone levels drop postpartum, prior to menses, and at menopause, while most patients receiving hormone therapy around menopause noted no change in their condition [39, 47–50].

Carlsten et al. have described how pregnancy and hormonal changes lead to improvement of psoriasis; they propose that estrogens have both an immunosuppressive and immunostimulatory property promoting a state of immune tolerance [51]. Estrogens have been shown to stimulate B-cell mediated immunity while suppressing T-cell mediated immunity; progesterone, being primarily immunosuppressive, downregulates the T-cell proliferative response and has been shown to be the key factor in immunosuppression [51–56]. Therefore, it has been hypothesized that high levels of progesterone would correlate with improvement of psoriatic symptoms [46]. It was observed that progesterone levels increased more dramatically during pregnancy compared with estrogen levels and it has been proposed by Carlsten et al. that the change in the estrogen-progesterone ratio produces an altered immunity [39, 51]. However, Murase et al. report on findings indicating that increased estrogen levels and especially increased levels of estrogen relative to progesterone correlate with improvement of psoriasis. They found that progesterone levels alone did not correlate with change in psoriasis and therefore it can be assumed that patients who experience an improvement of psoriasis have higher levels of estrogen relative to progesterone during pregnancy, whereas those who have lower ratio levels will remain unchanged or potentially worsen [13]. The estrogen concentration in peripheral blood gradually increases throughout the early to late stages of pregnancy, subsequently decreasing after parturition and eventually reaching nonpregnancy group levels within one month postpartum [57]. Weatherhead et al. found that half of their patients with psoriasis presented with a flare-up of symptoms within six weeks of delivery, which correlates with the previously mentioned observations implicating the role of hormones in psoriatic symptoms [58].

Dermatological changes occur in about 90% of pregnant women in one form or another and the associated skin changes may be either physiological (hormonal), changes in preexisting skin diseases, or development of new pregnancy-specific dermatoses. All of these conditions can be linked to either profound hormonal, vascular, metabolic, or immune changes occurring during pregnancy and treatment can be difficult [59]. Unfortunately, treating psoriasis in pregnant and lactating women presents a special challenge. Due to obvious ethical reasons, prospective randomized control trials have not been conducted in this patient population, although these patients do encounter new-onset psoriasis in addition to flares and may require treatment throughout their pregnancies [60].

## 4. Menopause

During menopause, endocrine disorders can be the cause of many skin diseases or conditions. Menopause, like pregnancy or menstruation, modulates the natural course of psoriasis [15, 61]. In perimenopause, many different hormonal changes occur and the onset of perimenopause or menopausal transition is marked by the end of menstrual cycle regularity and is associated with decreases in the production of ovarian inhibiting hormones [62]. During the early follicular phase of the menstrual cycle, slightly elevated but highly fluctuating follicle-stimulating hormone (FSH) levels may be observed. These levels gradually become consistently elevated into the late perimenopause and postmenopause, while estrogen and progesterone levels decrease and luteinizing hormone levels increase as the woman approaches menopause. The postmenopausal period is divided into early and late phases, marked by significant decreases in estrogen production, an overall state of hypogonadism, stability in the hypothalamic-pituitary-gonadal axis, and elevated FSH levels [63]. A decrease in estrogen during menopause is believed to be a major factor in the occurrence or exacerbation of psoriasis flare-ups in patients already suffering from psoriasis and it is believed that reduced estrogen levels lead to insufficient Th1 cell-mediated response inhibition, playing a major role in the pathogenesis of psoriasis. The study by Kanda and Watanabe has shown that *β*17 estradiol (E2) inhibits the production of IL-12 and TNF-*α*, reducing the ability of dendritic cells to present antigens and therefore to stimulate the synthesis of the anti-inflammatory cytokine IL-10 by T-lymphocytes, and also exhibits an inhibitory effect on the Th1-type immune response. Therefore, a fall in estrogen concentration in postmenopausal women can be attributed to the exacerbation of psoriasis. This finding may be useful since estrogen and/or progesterone may be potentially beneficial in the treatment of psoriasis [15]. In a study conducted by Mowad et al., menopausal women had an exacerbation of psoriasis in 48% of cases, while only 2% showed improvement; 27% of those observed noticed a link between psoriasis and hormonal changes [39]. Swanbeck et al. report on similar results and conclude that the late onset of psoriasis is more common in women than in men, suggesting that hormonal changes associated with menopause are a potential factor contributing to the development of psoriasis [64].

Generally, it can be assumed that high levels of estrogen have a rather regulatory and inhibitory effect on many components of the immune response, while low levels can affect it or even be stimulating [32, 36, 37]. A good example is the TNF-*α* molecule; the low estrogen concentrations typical of postmenopausal women have a stimulatory effect on the production of this cytokine, whereas high concentrations inhibit its synthesis, which could be crucial in the understanding of psoriasis in postmenopausal women [16, 32]. Therefore, it can be assumed that the decline in estrogen levels during menopause may be responsible for worsening of psoriasis [32].

## 5. Conclusion

The severity of psoriasis in a female patient may fluctuate with hormonal changes since psoriasis develops more frequently or gets worse at puberty, with another smaller peak at menopause. Often, there is a marked symptomatic improvement or even disappearance during pregnancy, only to reappear after childbirth. With such strong data linking hormones and psoriasis, estrogen and/or progesterone may be potentially useful in the treatment of psoriasis. Due to ethical reasons, clinical trials are not conducted in pregnant patients, although this population does encounter new-onset psoriasis in addition to disease flare-ups. Additional research is required before any conclusions can be drawn. It is important for the patient as well as the physician to be aware of the possible relationship between psoriasis and the hormonal phases throughout a woman's lifetime in order to effectively control or ease any symptoms that may arise.
